# Connectomics of Morphogenetically Engineered Neurons as a Predictor of Functional Integration in the Ischemic Brain

**DOI:** 10.3389/fneur.2019.00630

**Published:** 2019-06-12

**Authors:** Axel Sandvig, Ioanna Sandvig

**Affiliations:** ^1^Department of Neuromedicine and Movement Science, Faculty of Medicine and Health Sciences, Norwegian University of Science and Technology, Trondheim, Norway; ^2^Department of Neurology, St. Olav's Hospital, Trondheim University Hospital, Trondheim, Norway; ^3^Department of Pharmacology and Clinical Neurosciences, Division of Neuro, Head, and Neck, Umeå University Hospital, Umeå, Sweden

**Keywords:** neural networks, neuroengineering, cell reprogramming, disease modeling, multielectrode arrays, computational modeling, electrophysiology, cell therapy

## Abstract

Recent advances in cell reprogramming technologies enable the *in vitro* generation of theoretically unlimited numbers of cells, including cells of neural lineage and specific neuronal subtypes from human, including patient-specific, somatic cells. Similarly, as demonstrated in recent animal studies, by applying morphogenetic neuroengineering principles *in situ*, it is possible to reprogram resident brain cells to the desired phenotype. These developments open new exciting possibilities for cell replacement therapy in stroke, albeit not without caveats. Main challenges include the successful integration of engineered cells in the ischemic brain to promote functional restoration as well as the fact that the underlying mechanisms of action are not fully understood. In this review, we aim to provide new insights to the above in the context of connectomics of morphogenetically engineered neural networks. Specifically, we discuss the relevance of combining advanced interdisciplinary approaches to: validate the functionality of engineered neurons by studying their self-organizing behavior into neural networks as well as responses to stroke-related pathology *in vitro*; derive structural and functional connectomes from these networks in healthy and perturbed conditions; and identify and extract key elements regulating neural network dynamics, which might predict the behavior of grafted engineered neurons post-transplantation in the stroke-injured brain.

## Introduction

Stem cell therapy for ischemic stroke may extend the therapeutic window from the acute into the sub-acute and chronic stage. As such, it is a particularly interesting approach, considering that more than 80% of stroke patients are *de facto* not eligible for the standard clinical treatment options, i.e., thrombolysis or thrombectomy (1). Numerous animal studies have demonstrated the potential of stem cell therapy alone, or in combination with *in situ* tissue engineering strategies and/or pharmacotherapeutics, for promoting functional restoration after stroke. The rationale behind stem cell therapy is that it can promote functional recovery through a range of possible mechanisms of action, including neuroprotection, modulation of the inflammatory response, cell replacement, remyelination, tissue/vascular remodeling and *de novo* neurogenesis (2–8). Although promising and valuable in the quest of elucidating relevant recovery mechanisms at the experimental level, clinical translation of stem cell-based approaches tends to be largely confounded by significant challenges. These include poor graft survival and integration with the host tissue, which indeed is hardly surprising, given the intricacies inherent in aspiring to create safe, functional *in situ* biointerfaces that could effectively re-establish functional connectivity in multiple foci (9–12). As a result, numerous past and ongoing clinical trials of various stem cell-based therapies have largely failed to fully confirm preclinical findings by promoting significant and/or long-lasting gain of motor, sensory, or cognitive function in stroke patients (10, 11, 13–15).

Apart from the conceivable limitations of extrapolating findings from animal studies to the clinic, a substantial barrier to clinical translation is the lack of a thorough understanding of the underlying mechanisms determining cell graft behavior post-transplantation in the ischemic brain. This is further confounded by the intrinsic complexity of stroke lesions and, with regard to cell replacement and functional integration of the engrafted cells, also the lack of direct empirical evidence supporting neuronal replacement in human patients (1).

It is also worth considering the potential role of endogenous plasticity in response to ischemic injury, not least, the manner in which associated mechanisms may influence transplant behavior (16). Plastic responses after stroke can be demonstrated as neurogenic niche activation and cytogenesis in the ipsilesional brain hemisphere, as well as rewiring of surviving neural networks and recruitment of intact synapses contralateral, but also ipsilateral to the lesion (17–22). Such plastic responses and, not least, concomitant inflammatory processes triggered by brain ischemia, may conceivably play a significant role in determining functional outcomes after stroke (12, 23, 24). The underlying mechanisms regulating such responses are however unclear and, consequently difficult to harness in an appropriate or timely manner. Furthermore, the fact that intrinsic neuroplasticity can be adaptive, but also maladaptive, suggests that any attempt to harness it that fails to fully comprehend or suitably engage fundamental underlying mechanisms may inadvertently exacerbate lesion-induced deficits rather than contribute toward functional restoration.

Several questions therefore arise that warrantee rigorous discussion in the scientific community. In this review, we aim to contribute to this discussion by providing a concise overview of novel and emerging theoretical and methodological perspectives which may be significant for improving the robustness of pre-clinical studies with a view to clinical translation. Specifically, we propose the investigation of *in vitro* engineered neural networks in the context of connectomics and discuss how morphogenetic neuroengineering can be supported by advanced interdisciplinary approaches, including *in vitro* electrophysiology, microfluidics, and computational modeling, to obtain robust preclinical models that can promote our understanding of cell replacement therapy for stroke beyond the state-of-the-art.

## Morphogenetic Neuroengineering

The differentiation and development of projection neurons in the mammalian neocortex is regulated by tight molecular control, which determines and orchestrates sequential cell fate decisions over different temporospatial scales [reviewed in Greig et al. (25)]. An understanding of fundamental mechanisms regulating these processes led to the development of morphogenetic neuroengineering approaches based on controlled expression of transcription factors (26, 27). In 2002, a proof-of-principle study by the Göetz lab demonstrated that the Pax6 gene controls the neurogenic potential of radial glia, and also that its forced expression can induce astrocytes to a neuronal fate *in vitro* (28). A few years later, the same group showed that upregulation of Olig2 expression after brain injury, including focal ischemia, suppresses Pax6 expression, while *in vivo* transduction with a dominant-negative form of Olig2 in the acute and chronic phase after injury can induce neurogenesis in the neocortex (29).

A major barrier in morphogenetic neuroengineering with a view to clinical translation for cell replacement therapy was broken by Sinya Yamanaka's research. In a seminal paper in 2006, Yamanaka and Takahashi demonstrated conversion of adult somatic cells into induced pluripotent stem cells (iPSCs) after transduction with only four genes (Oct4, Sox2, Klf4 and c-Myc) (30). This method paved the way toward autologous sourcing and generation of theoretically unlimited numbers of stem cells applicable to a range of possible therapeutic approaches in regenerative medicine, including treatment of central nervous system (CNS) lesions, such as ischemic stroke.

Recent advances in cell reprogramming technologies have led to the development of efficient, reproducible, high-yield protocols for the generation of iPSCs and induced neural stem cells (iNSCs), as well as protocols for the generation of induced neurons (iNs) and specific neuronal subtypes, including spinal motoneurons, dopaminergic, cholinergic, and medium spiny neurons, and also cortical neurons, by direct conversion of somatic cells (31–37); reviewed in Gascon et al. (38). Such direct conversion protocols bypass the pluripotency state through forced expression of lineage-specific transcription factors regulating brain development and may also effectively preserve the age-related and epigenetic imprint of the cell (31, 39). Similar principles can be applied for direct reprogramming of glia, including astrocytes, NG2 glia, microglia, and pericytes, into neurons (40–44). Interestingly, a number of studies have also demonstrated *in vivo* reprogramming of resident brain and spinal cord astrocytes and endogenous neural progenitors into neurons (44–51).

Reprogramming through the iPSC/iNSC state or by direct conversion is highly relevant for cell-based therapies for brain ischemia. A number of studies have demonstrated the potential of iPSC and iNSC-derived neurons for cell replacement therapy in stroke (4, 52). Furthermore, neurons derived from immature progenitors and pluripotent cells have the potential to establish long-range target-specific functional connections in the adult mammalian CNS (49, 53–57) reviewed in Wuttke et al. (58).

However, unequivocal evidence of functional integration of iPSC/iNSC derived neurons in the ischemic brain post transplantation is scant, not least regarding the potential of these cells to establish long-term efferent connections in a target-specific manner (1). Tornero and co-authors were the first to show that cortical neuronal progenitors, differentiated from human fibroblast-derived iPSCs, survive after intracortical transplantation in the rat brain after stroke and also receive afferent inputs, as suggested by observed monosynaptic responses from the grafted neurons after stimulation of the intact cortical region adjacent to the graft (59). Furthermore, in a recent study, the same group demonstrated that transplanted iNSC-derived neurons can functionally integrate with the local thalamocortical circuitry and receive direct synaptic inputs from the relevant brain regions (60).

Taken together, such evidence suggests that the state-of-the-art morhogenetic neuroengineering approaches for the generation of neuronal types relevant for cell replacement therapy in stroke hold promise. Their potential for clinical translation, however, is not without caveats. A fundamental, currently unresolved, question as whether transplanted engineered neurons can promote improved functional outcomes after stroke is the extent to which they can morphologically and functionally integrate with the host tissue.

Several related questions arise: Do engineered neurons have the capacity to establish efferent connections, in addition to afferent ones, post-transplantation? If so, where in the brain are transplanted neurons likely to project and form synapses? How does the transplant respond to evolving stroke related pathology, including the spontaneous reorganization of the relevant neural circuitry after the injury, as well associated dynamic changes at the micro-, meso-, and macroscale? Is it possible to anticipate such responses and, importantly, predict whether functional integration of the transplanted neurons in the host brain will elicit adaptive or maladaptive processes?

## Connectomics of the Healthy and Lesioned Brain

### Adaptive and Maladaptive Neuroplasticity

To achieve therapeutic interventions for stroke beyond the state-of-the-art we need to consider stem cell therapy in the context of the extremely complex, highly interconnected structural and functional circuitry of the human brain, especially the altered, highly plastic, structure-function relationships triggered by an ischemic lesion. Endogenous neuroplasticity after stroke and its relevance for the functional integration of morphogenetically engineered transplants to elicit recovery of motor, sensory, or cognitive deficits as a result of brain ischemia, can be briefly discussed in the context of Hebbian and homeostatic mechanisms.

During development, the CNS is characterized by a high level of plasticity, where functional brain networks and circuits are shaped by Hebbian mechanisms, such as long-term potentiation (LTP) and long-term depression (LTD) (61, 62) constituting the synaptic basis of associative learning (63). These processes are regulated by evolutionarily conserved mechanisms and involve functional and structural changes which occur in a well-orchestrated spatiotemporal manner. However, once development has been completed, Hebbian mechanisms alone cannot explain how the mature brain maintains normal function. In the same manner, Hebbian laws *per se* cannot sufficiently explain plastic network responses, such as synaptic scaling (64, 65), which are observed following externally induced perturbations. This suggested that there may be another form of plasticity underlying such responses. Compelling experimental evidence over the last two decades supports that another form of plasticity, i.e., homeostatic plasticity, does exist (64, 66–69). Indeed, homeostatic plasticity operates in tandem with Hebbian plasticity along complex temporal and spatial scales and serves to maintain normal neural network function as well as regulate network responses to perturbation (67, 70).

It is well-documented that any major disturbance to normal neural network function tends to result in severe, permanent functional deficits, but also trigger adaptive and maladaptive network responses, which are demonstrated as changes in network structure and function (12, 18, 19, 66, 69–74). Such alterations may represent various underlying forms of neuroplasticity, which involve both homeostatic and Hebbian mechanisms (67, 75, 76), and which can be described as morphology-activity, (i.e., structure-function) relationships. Such relationships are highly complex and, as a result, poorly understood. However, elucidation of how different forms of plasticity may result in adaptive or maladaptive neural network responses and, thereby, how such processes may promote or hinder functional restoration after a perturbation such as stroke is of fundamental importance for our ability to achieve functional repair.

As mentioned earlier, plastic responses to an ischemic event include spontaneous functional recovery (77), rewiring of surviving neuronal networks and axonal ramification, and the recruitment of intact synapses post-lesioning (78), demonstrated as intra- and interhemispheric neural network remodeling (74). Furthermore, there is increasing consensus as to the likelihood that such responses are also influenced by evolving inflammatory processes (12). The underlying mechanisms regulating such responses largely involve structural as well as functional changes in brain circuits closely associated with the ones directly affected by stroke. These circuits can further be engaged and modified through experience-dependent plasticity (17, 79).

It is a reasonable assumption to make that there is a critical period of increased neuroplasticity after stroke, which can be harnessed to promote functional recovery, as exemplified in a number of preclinical as well as clinical studies (12, 17, 77, 80–84). Moreover, recent evidence suggests that recovery after stroke can be mediated through the engagement of neural networks in a functional hierarchy both in the contralesional as well as ipsilesional brain hemisphere and also upstream as well as downstream of the lesion (85). Furthermore, it is becoming increasingly evident that any cortical remapping that may occur after stroke is both activity dependent, but also based on competition (86).

### Neural Transplants as Part of the Brain Connectome

Where does cell replacement therapy fit in this context? To seek answers to this question, it is important to consider the behavior of engineered neural transplants in the ischemic brain in terms of connectomes (87, 88) i.e., the changing structure-function relationships and complex interplay between affected and intact neural ensembles in the brain in the context of evolving stroke pathology.

Highly relevant insights as to how the brain connectomes underpin the behavior of a neural system and determine functional outcome after a perturbation can be found in an excellent review by Fornito and co-authors (89). In this paper, the authors discuss how the highly interconnected, complex topology of the neural architecture of the brain, which is characterized by a fine, coordinated balance between regional segregation and specialization of function, but also highly precise functional segregation and integration of distal neuronal ensembles across multiple temporal scales, can shape the brain's response to perturbations (89–93). Specifically, brain network topology can determine not only disease progression, but also adaptive or maladaptive responses to pathological perturbations. By applying fundamental principles from graph theory, it is possible to extract key functional and structural elements of the brain's connectome that may explain or predict such responses (94).

According to graph theory, a neural assembly consists of key elements, namely nodes and edges, and as such, metrics that define properties of the graph can be applied to make inferences about the neural connectome, for example, as it is revealed by electrophysiological activity (92, 94–96). Nodes incorporate neurons, neuronal populations, and macroscopic brain regions, while edges determine structural, functional, or effective connectivity and, as a result of the latter, the directionality of internodal neural communication. In this manner, structural connectivity can be indicative of morphology-activity relationships between neurons, whereas functional connectivity, which is independent of the physical proximity of the neurons, provides a temporal correlation of their activity. On the other hand, effective connectivity can determine whether there is a causal relationship between the activity of different neurons (94). Topological properties of particular interest include economical wiring, hierarchical modularity, small-world organization enabling functional segregation, integration and specialization through high network clustering and characteristic short path lengths (93, 95–100), as well as highly-connected network hubs. Connections between hubs can be describes as rich club, i.e., central core connections, if they extend over large anatomical distances and link distinct functional systems (101). As such, rich club connections mediate high-volume network traffic and integrated brain function.

### Connectomics of Adaptive and Maladaptive Responses to Brain Ischemia

**Figure 1:**
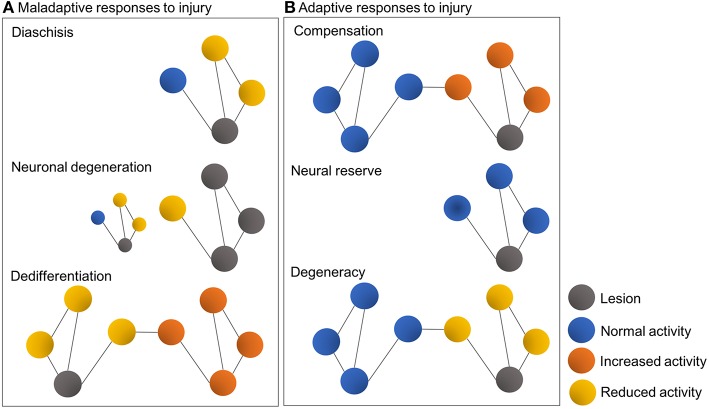
Schematic representations illustrating typical maladaptive **(A)** and adaptive responses **(B)** to brain injury in the context of connectomes and graph theory. Colored circles represent nodes, while lines represent interconnecting edges [Figure modified from Fornito et al. (89)].

As a result of high interconnectivity of neural networks and circuits, the damage induced by lesions such as focal cerebral ischemia dissipates in the brain and progressively spreads to affect distal loci (81). This diaschisis phenomenon constitutes a maladaptive response and may explain the deafferentiation and aberrant synchronization observed in brain regions remote from the one directly affected by the stroke (74, 89, 92). Excitotoxicity thus plays a central part in the damage sustained to these remote nodes after a stroke. Associated maladaptive responses include anterograde and retrograde neuronal degeneration, for example, as a result of neuronal loss, excitotoxicity and impaired axonal transport, but also dedifferentiation. Apart from loss of tissue at the lesion site, neuronal degeneration involves structural deterioration over time of nodes connected to the primary lesion site, while dedifferentiation refers to the gradual loss of specialized function in the affected brain region, followed by increased, non-specialized activity in associated nodes (89, 92) (Figure 1A).

On the other hand, adaptive network responses include lateralization, i.e., compensation for lost function by increased structure-function interactions and the engagement of intact neuronal ensembles within the affected system, or within other systems. Other adaptive responses may include neural reserve, a process by which remaining intact tissue in the immediately affected region can sustain the previous level of behavior, and also degeneracy, where a second system, without demonstrating apparent changes, can adequately support the activity of the affected one (89, 102). Thus, the effects of a perturbation critically depend on the topological centrality and degeneracy of the affected region, as pathology of central regions can exacerbate maladaptive responses, whereas degeneracy can facilitate adaptive ones (89, 95, 96). By the same token, spontaneous or induced gain of function after stroke may imply partial restoration of neural networks previously involved in impaired function or, alternatively, a compensatory or substitution mechanism, which is contingent on the recruitment of other networks (74) (Figure 1B).

However, in any perturbed system, not least, the ischemic brain, which is characterized by highly complex pathophysiological mechanisms and associated cellular and molecular cascades, such responses are neither unequivocal, nor mutually exclusive. Furthermore, as mentioned earlier, the resilience of the perturbed system and the manner in which it may reconfigure its structure-function relationships in response to the lesion largely depends on the topological substrates being affected. It follows that a neural transplant, effectively, a new topological element (likely, a node) within the local circuitry, becomes another integral component in the system's connectome. As such, the input and output functions of the transplant node are shaped by other key elements of the connectome, as for example, its interactions with other nodes, in an interdependent, reciprocal manner.

### Assessing the Potential of Morphogenetically Engineered Neurons to Become Integrated Within the Connectome

What are the exact processes that determine whether and/or to what extent neurons engineered *in vitro* (and by the same token, *in vivo*), have the capacity to become part of the local connectome in a manner that may promote gain of function after stroke?

Detailed study and elucidation of relevant mechanisms in stroke patients is not feasible and is thus *de facto* limited to methods and analyses based on indirect and often incomplete observations or measures. Relevant research must therefore rely on robust preclinical models. Notwithstanding various excellent studies employing cell replacement strategies, coupled with cutting-edge methods for monitoring and assessment of functional integration of the engineered neurons in experimental stroke animals (16, 60), there is currently no unequivocal evidence supporting that these neurons become fully integrated into the ischemic brain. As a result, pertinent questions remain unanswered, especially with regard to the intrinsic capacity of engineered neurons to engage with local circuitry, whether we can predict their responses to evolving pathology, as well as determine whether their morphological and functional integration into the host brain will elicit adaptive or maladaptive responses and, ultimately, promote repair or inadvertently exacerbate the lesion-induced deficits. We may start addressing such questions by combining *in vitro* morphogenetic neuroengineering with electrophysiology studies and computational modeling.

### Reductionist *in vitro* Paradigms That Capture Complex Network Dynamics in Healthy and Perturbed Conditions

State-of-the-art platforms for the study of *in vitro* neural networks include microelectrode arrays (MEAs). MEAs represent a breakthrough in *in vitro* electrophysiology platforms, as they enable direct observation of evolving network dynamics through extracellular recordings, as well as disease modeling and modulation of network activity by electrical, chemical, opto- or chemogenetic manipulation to selectively perturb network function or engage its plasticity (94, 103–111).

This approach largely exploits intrinsic attributes of *in vitro* neural networks, including emergence/morphogenesis, as a result of cell behaviors driven by local cell-cell interactions, and self-organized criticality (SoC) (112). A universal characteristic of *in vitro* cultured neurons, irrespective of source (i.e., rodent or human) or specific subtype, is that they self-organize into functional complex networks. This spontaneous process, which occurs in the absence of any external instructive influence (113), involves the network's gradual transition through distinct morphology-activity states underscored by increasing structural and functional complexity as a result of emerging neuronal activity, axonal elongation and pathfinding, synapse formation and pruning (113, 114). Over time, the network gains morphological and functional maturity, ultimately reaching SoC, a critical state characterized by a fine balance between excitation and inhibition (113, 115–118). Once at the SoC state, the neural network exhibits neuronal avalanches, i.e., characteristic cascades of activity defined by rich, stable spatiotemporal patterns and power-law distributions of variables (114, 119). Criticality has been closely, yet not exclusively, associated with self-organization (112, 120–124).

How does SoC displayed by *in vitro* neural networks relate to normal or impaired brain function? The idea that the brain operates in a near-critical state is not new (119, 125, 126), yet the presence of SoC states in the brain has, to a certain extent, been the subject of debate until relatively recently (119, 127–129). However, emerging evidence, including findings from magnetoencephalography (MEG) studies in human subjects, strongly supports the presence of SoC in the brain, not only as a mechanism underlying spatially local or internodal measures of brain activity, for example, as relevant for cortical function, but also as a global phenomenon (123, 130).

This suggests that within the brain connectome, SoC states play a putative role in information transmission and processing, including segregation and integration of function along different spatiotemporal scales (114, 123, 124, 131). As such, critical states in the brain emerge from the complex underlying morphology-activity relationships shaping the system's behavior and are stipulated to underscore adaptation processes in the context of neuroplasticity. It is currently unknown how changes in network organization over different spatiotemporal scales, for example, as a result of ischemic insult, may affect such states (114).

**Figure 2:**
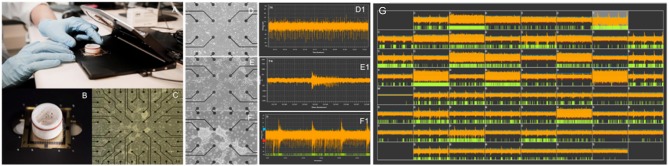
Evolution of spontaneous electrical activity in a morphogenetically engineered neural network on a 60-electrode MEA. **(A)** Standard *in vitro* electrophysiology platform (Multi Channel Systems, Germany). **(B)** Standard planar 60-electrode MEA with protective ring for long-term neuronal culture. **(C)** Overview image showing a neural network on a 60-electrode MEA. Neuronal clusters appear as dense white regions on the MEA. **(D–F)** Self-organization of engineered neural networks on an MEA over time and corresponding development of spontaneous activity shown as tonic firing **(D1)**, unsynchronized bursts **(E1)**, and pacemaker bursts **(F1)** at 20, 28, and 40 days *in vitro*, respectively. **(G)** Shows the mature neural network at >45 days *in vitro*.

*In vitro* biological substrates that combine morphogenetically engineered neurons with MEAs can be used to obtain insights into such mechanisms. For example, neural avalanches can be observed and recorded as synchronous local field potential (LFP) spikes across multiple electrode locations. Dependent on the number of embedded recording electrodes available on the MEA, which may range from a few dozen (typically 60–120 electrodes) to several thousand, the observed LFPs may arise from neuronal clusters, effectively individual nodes within the network, but also represent single-cell activity, as in the case of very high-density (>11,000 electrodes) complementary metal-oxide semiconductor (CMOS) integrated circuit MEAs (118). This effectively means that the derived neural substrates can faithfully recapitulate fundamental aspects of neural network behavior, thus enabling the study of complex network dynamics in reductionist *in vitro* paradigms. Maintenance of the relevant MEA-based neural networks in optimal culture conditions enables long-term monitoring of network behavior up to several weeks or months (132) (Figure 2).

#### Verification of Functionality

In light of the above, an immediate application of *in vitro* morphogenetically engineered neurons on MEAs can be the validation of the neurons' functionality prior to transplantation in the ischemic brain. This is a crucial but often overlooked parameter, given that fundamental intrinsic properties of engineered neurons, such as self-organization, spontaneous firing activity, or capacity to reach SoC states, can be expected to provide a gauge as to their potential to survive and integrate with the host tissue post-transplantation. As such, electrophysiological recordings from engineered neurons on MEAs is a highly complementary method to transcriptional analyses of such neurons after reprogramming and should constitute an integral part of their characterization before proceeding to *in vivo* studies.

Importantly, apart from verification of the inherent capacity of *in vitro* engineered neurons to self-organize into functional networks, reductionist *in vitro* paradigms such as MEA-based biological substrates also constitute a powerful tool for assessing the behavior of these networks in healthy and perturbed conditions by enabling the study of their connectomes.

Several recent studies have demonstrated that *in vitro* neural networks, including human engineered neuronal cultures, demonstrate physiological maturation over time suggestive of complex underlying structure-function relationships (133–138). Functional maturation can be further enhanced typically by culturing neurons on a feeder layer of astrocytes (139, 140), or by adapting the differentiation protocol to generate both neurons and astrocytes (141), thus optimizing neuronal polarization, axonal and dendritic arborization, and synapse formation in the derived networks (142–144). In this manner, *in vitro* neural networks recapitulate fundamental topological properties of the brain, including key features mentioned earlier, such as hierarchical modularity and small-world organization (98, 100).

Various methods for MEA data analysis can be applied to make inferences about the functional, structural or effective connectivity of the network, as well as its dynamical or critical states (94, 145). Such inferences are particularly relevant for obtaining insights as to the state of a neural network (including sub-critical and super-critical states) (113, 115, 146–148) but also for identifying evolving pathology, and predicting the manner in which the network will respond, i.e., adaptive or maladaptive.

#### Mimicking Stroke-Related Pathology

As mentioned earlier, one of the fundamental yet unanswered questions pertaining to the functional integration of *in vitro* engineered neurons in the ischemic brain post-transplantation is lack of unequivocal evidence from *in vivo* stroke models as to the ability of these grafts to extend axons and form efferent connections with appropriate targets. A corollary to the above is the capacity of such grafts to become incorporated in the local circuitry while pathophysiological processes of the injury and/or spontaneous plasticity of the host brain are in progress. MEA-based neural substrates provide an excellent platform for mimicking key aspects of such processes in a controlled manner.

There are currently no reported studies in the literature investigating stroke-related pathology in human engineered neural networks using MEA-based platforms. However, a number of reports have demonstrated the utility of these platforms for CNS disease modeling, often in the context of neurodegenerative disorders, epilepsy, toxicology, and drug screening, as well studies of neural network plasticity and response to selective modulation (108, 149–151).

Beyond the state-of-the-art modeling of engineered neural network responses to stroke may be achieved by the incorporation of defined topography and/or dimensionality in the MEA-based substrate. For example, a host of different biological and synthetic scaffolds can be applied for bioengineering of *in vitro* neural networks in three-dimensional (3D) cultures as opposed to the conventional two-dimensional (2D) ones (152–155). Such systems, which may also combine the use of a scaffolding substrates with 3D- rather than planar 2D-MEAs, can help recapitulate structure-function relationships shaped by cell morphology, cell-to-cell interactions, and axonal outgrowth in all directions within the extracellular microenvironment. In this manner, the derived *in vitro* connectomes can better approximate the micro-, meso-, and macroscale dynamics of the brain, thus enabling self-organized neural network behavior of increased complexity, compared to 2D systems (150, 156, 157). Importantly, 3D substrates are compatible with co-culture of neurons with other relevant cell types, including astrocytes and microglia, thus adding to the physiological relevance of the assay (142, 144, 158–162).

Hence, the 3D configuration can be used to assess emerging morphology-activity relationships in unperturbed engineered neural networks *in vitro*, but also to study their responses to stroke related pathology, as for example, after oxygen-glucose deprivation or induced excitotoxicity. The versatility and relevance of these platforms for *in vitro* connectomics of engineered neurons, can further be enhanced with the incorporation of microfluidics devices, which enable *in vitro* culture of modular neuronal ensembles with definable connectivity at the micro- and nanoscale level.

Custom-designed microfluid devices can be fabricated using a variety of methods including etching techniques, photo- and e-beam lithography, embossing, replica molding, and laser photoablation (163). Such systems enable the culture of *in vitro* neural networks by confining neuronal somata within designated compartments, i.e., nodes, and controlling afferent and efferent inter-nodal connectivity by predefined, directional axonal outgrowth through micropatterned channels only permissible to neuronal axons and neurites (164). As such, microfluidic devices can be superior to conventional culture systems as they enable spatial and temporal control of the neuronal microenvironment (163). Importantly, such microfluidic devices can be interfaced with MEAs, thus enabling longitudinal monitoring of intra- and internodal network dynamics in normal and perturbed conditions.

These integrated *in vitro* platforms may thus constitute a powerful tool for the investigation of the connectomics of morphogenetically engineered neural networks alone, or in co-culture with other relevant cells, including different relevant neuronal subtypes, glia, and microglia (142, 158, 165, 166). A potential application of multi-nodal microfluidic devices integrated with MEAs is to test whether compartmentalized engineered neurons can form integrated multi-nodal functional networks which demonstrate efferent, as well as afferent connectivity. Such studies can thus help address a pertinent, yet unelucidated question regarding engineered neural networks, namely, whether they can form efferent synapses. Furthermore, the topology and molecular/flow gradients of the microfluidic platform can be exploited to induce disease-related pathologies in a selective or controlled manner and monitor dynamic intra- and inter-nodal responses over time (167, 168). Added microtopography features, such as the incorporation of synaptic chambers (169), enable monitoring of axons and synapses at cross-sections between nodes, and also facilitate added manipulations, as for example axotomy (170, 171). Depending on electrode alignment in the MEA interface, such microfluidic designs can also enable monitoring of spike propagation along axons between nodes (172), as well as selective electrical stimulation.

#### Distinctive Advantages of *in vitro* Models

Clearly, advanced *in vitro* modeling platforms are highly versatile and can recapitulate complex morphology-activity relationships underpinning brain function (173–175). Such *in vitro* systems afford unique scope for mimicking stroke-related pathology and determining dynamic behavior of engineered neurons in the context of functional integration. Thus, although *in vitro* models are reductionist in nature, they are by no means simplistic and, compared to *in vivo* models, they enable specific manipulations and observations in a highly controlled manner. Especially with regard to connectomics studies, *in vitro* models have the distinctive advantage of allowing the study of emerging, dynamic network behavior at the micro- and mesoscale across large populations of neurons in different assemblies, including their internodal interactions (166, 176). Advanced *in vitro* models are thus highly complementary to *in vivo* ones, constituting valid alternatives and providing valuable insights that can instruct the design of *in vivo* stroke transplantation studies with improved predictive validity for clinical translation. As such *in vitro* modeling platforms are also aligned with the 3R principles (i.e., Replace, Reduce, Refine) pertaining to the use of experimental animals.

### Deep Learning; Making Inferences and Predictions About Behavior

Notwithstanding the tremendous potential afforded by these advanced *in vitro* platforms for studying engineered neural networks in the context of stroke modeling, it must be emphasized that MEA-based electrophysiology tends to generate very high volumes of data (typically, a standard 10 min recording from a 60-electrode MEA can generate 0.5 GB of data; indicatively, the corresponding data volume increases to 1,5 GB when using a 4,096-electrode MEA). As a result, the study of connectomes, which requires extraction of functional, structural and effective connectivity and, by the same token, inference about the underlying neural network state, or prediction about its dynamic responses to stroke-related pathology, including anticipated behavior post-transplantation *in vivo*, cannot be achieved with standard software analysis methods. Advanced computational modeling applying deep learning principles is thus necessary.

As mentioned earlier, connectomics analyses may apply graph theory and relevant theoretical models to determine underlying structure-function relationships within a neural network and infer its critical states (94, 109, 116, 117, 172). For example, advanced computational modeling may apply principles from cellular automata (CAs) or random Boolean networks (RBNs), which examine the relationships between constituent key elements of a system based, respectively, on proximal or random connections (177). Furthermore, machine learning or deep learning approaches, such as artificial neural networks (ANNs) and artificial recurrent neural networks (RNNs), are highly relevant computational models for MEA data analysis. Such models take inspiration from biological neural networks (BNNs). Briefly, in these models the neurons, i.e., the computing elements of the system, are arranged in layers through which information flows in a feed-forward manner. Interestingly, temporal dynamics of the systems can be captured by RNNs by assigning recurrencies (cycles) within the connection topology of the neurons in the system. As a result, the system possesses a memory of previous inputs; in other words, the activation state of the network at a given time represents a function of its previous activation states. As such, an RNN can be seen as an untrained reservoir of dynamics that operates in a near-chaotic state. This enables control of the system by selecting only a single linear redout layer for training (178).

RNNs are thus well-suited to capturing dynamic network behavior of *in vitro* neural networks, including their critical states. These attributes of RNNs make them an excellent computational paradigm for the investigation of mechanisms by which neuronal populations, including engineered neural networks, solve various computational problems in healthy and perturbed conditions (178–182). In this way, we can start addressing fundamental questions that can help determine the functional capacity of engineered neurons. Moreover, such models can be applied to decipher as well as predict the behavior of engineered neurons in response to stroke-related pathology *in vitro* and also post-transplantation *in vivo*. Importantly, the relevant computational models can be applied to make inferences about underlying critical states, including spontaneous and induced plasticity of these networks.

Finally, closed-loop hybrid systems are highly relevant in this context as they can be used for advanced simulations of spontaneous and induced plasticity, including bi-directional learning. For example, by interfacing an engineered neural network on an MEA with a simulated agent (i.e., computer hardware) we could elicit real-time agent behavior powered by the neural network using reservoir computing (183). Furthermore, by selecting a pacemaker node within the engineered neural network as the target for electrical stimulation, we were able to alter the pacemaker activity in a manner suggestive of LTP, and at the same time demonstrate learning behavior in the simulated agent (183). Other reported studies have also demonstrated the capacity of embodied neural network cultures for goal-directed behavior and learning (184–186). Such approaches are highly promising for the future development and application of relevant experimental paradigms for *in vitro* and *in silico* assessment of the endogenous as well as induced plasticity of engineered neural network, as for example, in terms of guided closed-loop neuromodulation approaches post-transplantation.

## Conclusions

In this review, we have discussed how the combination of interdisciplinary methods and theoretical principles can help develop robust pre-clinical experimental paradigms that can help validate the functional capacity of morphogenetically engineered neurons as well as provide significant insights as to their potential for promoting functional outcomes after transplantation in the stroke lesioned brain. This integration of approaches is both timely and necessary and can be expected to make significant contributions toward the safe translation of stem cell therapy in the clinic.

## Author Contributions

AS and IS contributed equally to all aspects pertaining to the design and preparation of this manuscript, including idea for the review, literature search and selection, writing, editing, and finalizing the manuscript.

### Conflict of Interest Statement

The authors declare that the research was conducted in the absence of any commercial or financial relationships that could be construed as a potential conflict of interest.
